# Gastric Hematoma Secondary to Heterotopic Pancreas of the Stomach: Unexpected Cause of Upper Gastrointestinal Bleeding

**DOI:** 10.7759/cureus.26175

**Published:** 2022-06-21

**Authors:** Abdur R Rubel, Vui H Chong

**Affiliations:** 1 Department of Medicine, Pengiran Muda Mahkota Pengiran Muda Haji Al-Muhtadee Billah (PMMPHAMB) Hospital, Tutong, BRN; 2 Department of Medicine, Raja Isteri Pengiran Anak Saleha Hospital (RIPAS) Hospital, Bandar Seri Begawan, BRN

**Keywords:** helicobacter pylori, gastrointestinal bleeding, ectopic pancreas, intramural hematoma, gastrointestinal hemorrhage

## Abstract

Upper gastrointestinal bleeding remains a common cause of hospitalizations and common causes include peptic ulcer disease, esophageal and gastric varices, and malignancies. Infrequently, rare and unexpected causes are encountered. Initial management is generally the same regardless of the cause that is focused on hemodynamic stabilization followed by endoscopy to assess and treat the cause of bleeding. We report a case of a 19-year-old female who presented with upper gastrointestinal bleeding and endoscopy showed a submucosal hematoma secondary to gastric ectopic pancreas or heterotopic pancreas. She was also treated for *Helicobacter pylori *infection. She was managed medically and was discharged without further recurrence of bleeding.

## Introduction

Upper gastrointestinal bleeding remains a common cause of hospitalizations. Common causes include peptic ulcer disease, esophageal and gastric varices, and malignancies [1-3]. Other causes include erosive gastritis, esophagitis, Mallory-Weiss tear, and less common, a Dieulafoy’s lesion (large caliber protruding blood vessel without significant ulceration) [1-3]. Infrequently, rare and unexpected causes are encountered such as bleeding from gastric heterotopic pancreas (HP). The initial management is generally the same regardless of the cause that is focused on hemodynamic stabilization followed by endoscopy to assess and treat the cause of bleeding. We report an interesting case of gastric hematoma secondary to gastric HP that was successfully managed with medical treatment.

## Case presentation

A 19-year-old female with thalassemia minor (trait) presented with dyspepsia that was associated with nausea, retching, and vomiting of approximately 200 ml of semi-fresh blood. There was no history of nonsteroidal anti-inflammatory drugs (NSAIDs) use. She was otherwise hemodynamically stable (pulse rate was 88/min and blood pressure was 110/70 mmHg) and had mild epigastric tenderness on deep palpation. Serum hemoglobin was 9.9 g/dL (normal range: 12-16.0 g/dL). Serum amylase and coagulation profile (prothrombin time) were normal. Mallory-Weiss tear was suspected and she was started on intravenous omeprazole (80 mg bolus followed by 8 mg/h), intravenous metoclopramide (10 mg three times a day), and intravenous fluid. After fluid resuscitation, serum hemoglobin was repeated and this had dropped to 7.3 g/dL. Blood transfusion was then initiated and she was given a total of two units of packed cells.

**Figure 1:**
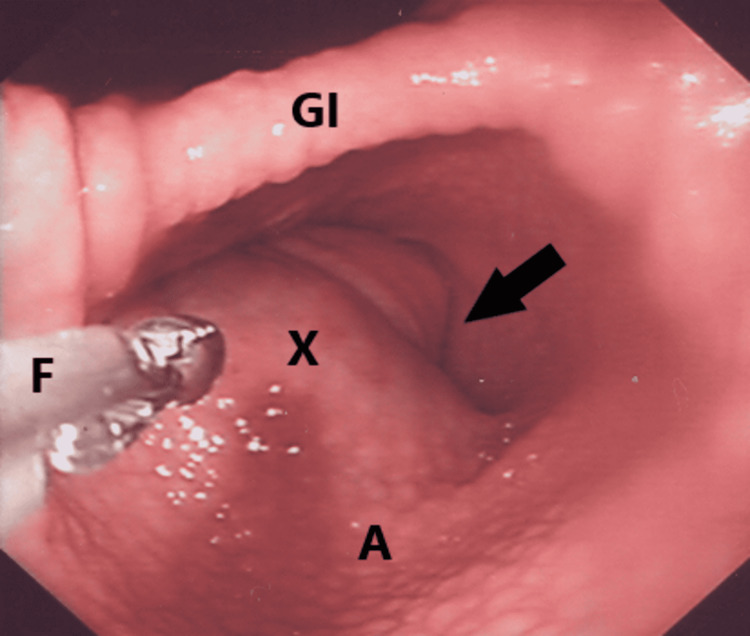
Upper gastrointestinal endoscopy of the patient. Endoscopy shows the antrum with a submucosal mass (indicated by X) on the greater curve with a bluish tinge pointing towards the pylorus (cannot be seen indicated by black arrow). The mucosal of the antrum greater curve has a granular texture (A). Forceps (F) and gastric incisura (GI).

Upper gastrointestinal endoscopy showed a large submucosal mass in the antrum with the tip pointing towards the pylorus (Figure 1). The mass was soft in texture and with forceps manipulation, a punctum with erosions could be seen at the tip of the lesion. There was no active bleeding noted but with forceps probing, slight oozing was noted from the punctum. No further attempt was made to manipulate the mass for concern of exacerbation of bleeding. The gastric mucosa was also noted to be nodular. The esophagus, duodenum, and fundus were otherwise normal. *Helicobacter pylori *(*H. pylori*) was positive on rapid urease test and later on histology.

**Figure 2:**
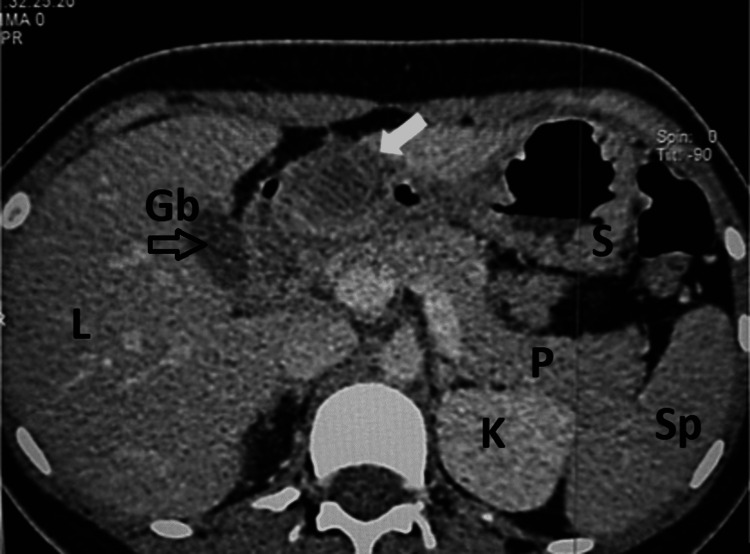
Axial computed tomography (CT) scan of the abdomen. The image shows a cystic mass in the antrum (white arrow) corresponding to the lesion seen on endoscopy. Liver (L), gallbladder (Gb indicated by open black arrow); body of stomach (S), spleen (Sp), pancreas (P), and left kidney (K).

A computed tomography scan showed a submucosal cystic 3.9 cm mass with the density of blood, consistent with a gastric intramucosal hematoma (Figure 2). 

**Figure 3:**
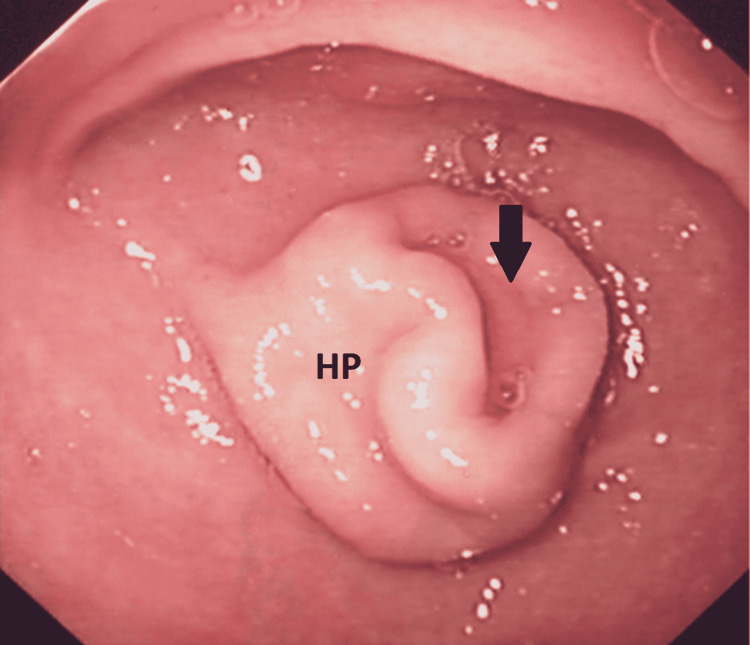
Repeat endoscopy was done after four weeks. The image shows a tubular structure in the antrum of heterotopic pancreas (HP) corresponding to the location of the submucosal mass seen in the first endoscopy. Opening of the HP located within the depression is indicated by the black arrow.

As her symptoms improved and serum hemoglobin improved to 10.1 g/dL, omeprazole was converted to oral formulation on day three of admission. *H. pylori* eradication therapy (omeprazole 20 mg twice daily, amoxicillin 1000 mg twice daily, and clarithromycin 500 mg twice daily) for a total of 14 days was then started. She was discharged on day four after admission. Repeat endoscopy was done four weeks later and this showed resolution of the hematoma. A tubular structure with a central umbilication was seen on the greater curvature in the antrum where the hematoma was previously located (Figure 3).

These findings and the location of the abnormality were consistent with endoscopic diagnosis of gastric ectopic pancreas or HP of the stomach. The mucosal nodularity seen previously had also resolved. Repeat *H. pylori* check was negative. She remained well on follow-up without any further symptoms or recurrence of bleeding. 

## Discussion

HP is an uncommon embryologic abnormality and is defined as aberrant pancreatic tissue separated anatomically from the main body and blood supply of the pancreas [4]. HP can have either exocrine or endocrine tissue, or both. HP is considered uncommon with reported rates ranging from as low as 0.6% in endoscopic studies to as high as 16% in autopsy studies [4,5]. There are slightly more men affected (61%) and the mean age of symptoms presentation was reported to be 43 years [4]. HP can be found in any part of the gastrointestinal tract. Up to 75% are found in the stomach, duodenum, and ileum, with stomach accounting for between 25% and 37% [5]. In a large series of 934 symptomatic patients, 59% of lesions were located in the stomach and 41% in the duodenum [4]. Generally, for stomach HP, 90% are located on the greater curvature of the antrum within 6 cm from the pylorus, appearing as a mucosal bulge with a central umbilication or punctum [6]. However, it can be located in any part of the stomach and without the typical features, especially for smaller lesions.

Most HP, regardless of the location are asymptomatic and are diagnosed incidentally. However, some can manifest with clinical symptoms and complications such as pancreatitis, gastric outlet obstruction, and even malignant transformation [4,5,7,8]. In a large single-center series that included 29 patients with upper gastrointestinal HP, only six (20.9%) had clinical symptoms [4]. However, a literature review of 232 publications (N=1762 patients) identified 934 (53%) patients with gastric or duodenal HP who had reported symptoms [4]. This study categorized clinical presentation/manifestations into four main categories. A majority presented with any abdominal pain (67%). Of patients with abdominal pain, 48% (n=445) had dyspepsia only (category 1), 28% (n=260) also had pancreatitis (category 2), 9% (n=80) also had gastrointestinal bleeding (category 3), and 9% (n=80) also had gastric outlet obstruction (category 4). There was a smaller group of patients presenting with other symptoms (jaundice and biliary obstruction due to ampullary lesions, perforations, fever, diarrhea, abscess, carcinoid syndrome, and dysphagia) [4]. Most patients were treated with surgical or endoscopic resection with 85% reporting resolution or improvement of their symptoms [4].

Gastrointestinal bleeding of any cause is potentially life-threatening. Bleeding from gastric HP has been reported to range from 7% to 17.5%, with a pooled analysis rate of 9% [4]. Bleeding is believed to the related to ulcerations of the gastric mucosa overlying the HP. In our case, the patient had erosions at the punctum of the HP. Another proposed mechanism involves chronic inflammation from HP that results in edema and congestion of submucosa [4]. Congestion of the fragile vasculature in the submucosa may lead to bleeding and later evacuate into the gastric lumen [4]. In our case, *H. pylori* infection might have been contributory.

The main difficulty with the management of HP is making a diagnosis prior to planning treatment and differentiating it from other submucosal lesions. For definitive diagnosis of HP, histology is required. However, normal biopsies are often nondiagnostic due to location of HP; submucosa (73%), muscularis (17%), and subserosa (10%) [5]. Use of modified techniques such as mucosal cutting to allow access to deeper tissue sampling has been advocated [9]. Use of endoscopic ultrasound (EUS) with or without fine needle sampling is another method with sensitivity ranging between 80% and 100% [5]. Noninvasive imaging studies have been reported to differentiate gastrointestinal HP from other gastrointestinal sub-epithelial tumors [10]. Characteristics features such as mucosal bulge with umbilication and antral greater curvature location have been shown to be diagnostic in older barium studies with sensitivity and specificity of 87.5% and 71.4%, respectively [5]. These features have also been reported for CT and endoscopy [10]. A review of histology confirmed HP displayed characteristic features on imaging; submucosal masses, ill-defined lesions with an endoluminal growth pattern, bright enhancement similar to the normal pancreas, surface dimpling, and low intralesional attenuation on CT scan and endoluminal, submucosal mass, typically with central umbilication on endoscopy [10]. In the presence of these typical features, a diagnosis of gastric HP can be reliably made with endoscopy. In our case, a diagnosis of HP was made based on the typical features of gastric HP on endoscopy. We did not do other imaging studies as endoscopy already showed typical features of gastric HP. Among these, EUS would have been preferred. However, this facility was not available in our setting. 

Resection has been advocated for symptomatic cases. However, this should also depend on symptoms manifestations, and concerns. To date, cases that have been treated with resection, either endoscopically or surgically, were done to manage complications or concern for underlying neoplasms. Often, diagnoses were only made after histological examination of resected specimens [4]. In our case, although discussed, we did not consider resection as the patient had recovered and opted for monitoring and intervention only if significant symptoms or complications arise. The main differential diagnoses were submucosal lesions, either benign or malignant. In our case, there was no concern of underlying malignancy given the patient's age, resolution of symptoms, and remaining well on follow-up. Based on one of the author's experiences with five cases of gastric HP, all diagnosed incidentally on endoscopy with similar features and locations, none of the patients had developed any symptoms related to this abnormality or had developed complications on follow-up. Similarly, this has been reported by others after follow-up of between 3 years and 13 years [11]. Regardless, follow-up has been recommended [11].

## Conclusions

HP is a congenital abnormality that is mostly asymptomatic and encountered incidentally during endoscopy or surgery. Symptomatic cases of HP may manifest with nonspecific symptoms but complications such as bleeding, pancreatitis, gastric outlet obstruction, and rarely malignant transformation have been reported. Therefore, resection has been advocated. However, for asymptomatic or those with symptoms that have resolved as in our case, whether resection is indicated remains a debate. This case highlights an uncommon cause of upper gastrointestinal bleeding from HP in the stomach that manifested as an intramural hematoma and was successfully managed with medical treatment. In this case, *H. pylori* might have contributed to the upper gastrointestinal bleeding.
